# Homology Modeling of Human γ-Butyric Acid Transporters and the Binding of Pro-Drugs 5-Aminolevulinic Acid and Methyl Aminolevulinic Acid Used in Photodynamic Therapy

**DOI:** 10.1371/journal.pone.0065200

**Published:** 2013-06-07

**Authors:** Yan Baglo, Mari Gabrielsen, Ingebrigt Sylte, Odrun A. Gederaas

**Affiliations:** 1 Department of Cancer Research and Molecular Medicine, Faculty of Medicine, Norwegian University of Science and Technology, Trondheim, Norway; 2 Medical Pharmacology and Toxicology, Department of Medical Biology, Faculty of Health Sciences, University of Tromsø, Tromsø, Norway; Children's Medical Research Institute, Australia

## Abstract

Photodynamic therapy (PDT) is a safe and effective method currently used in the treatment of skin cancer. In ALA-based PDT, 5-aminolevulinic acid (ALA), or ALA esters, are used as pro-drugs to induce the formation of the potent photosensitizer protoporphyrin IX (PpIX). Activation of PpIX by light causes the formation of reactive oxygen species (ROS) and toxic responses. Studies have indicated that ALA and its methyl ester (MAL) are taken up into the cells via γ-butyric acid (GABA) transporters (GATs). Uptake via GATs into peripheral sensory nerve endings may also account for one of the few adverse side effects of ALA-based PDT, namely pain. In the present study, homology models of the four human GAT subtypes were constructed using three x-ray crystal structures of the homologous leucine transporter (LeuT) as templates. Binding of the native substrate GABA and the possible substrates ALA and MAL was investigated by molecular docking of the ligands into the central putative substrate binding sites in the outward-occluded GAT models. Electrostatic potentials (ESPs) of the putative substrate translocation pathway of each subtype were calculated using the outward-open and inward-open homology models. Our results suggested that ALA is a substrate of all four GATs and that MAL is a substrate of GAT-2, GAT-3 and BGT-1. The ESP calculations indicated that differences likely exist in the entry pathway of the transporters (i.e. in outward-open conformations). Such differences may be exploited for development of inhibitors that selectively target specific GAT subtypes and the homology models may hence provide tools for design of therapeutic inhibitors that can be used to reduce ALA-induced pain.

## Introduction

Photodynamic therapy (PDT) is an innovative treatment modality for cancer that involves systemic or topical administration of a photosensitizer pro-drug, or the photosensitizer itself, and activation of the photosensitizer by light of appropriate wavelengths, resulting in generation of reactive oxygen species (ROS) and toxic responses [1]–[3].

One commonly used PDT pro-drug is 5-aminolevulinic acid (ALA). Topical (dermal) administration of ALA or the ALA methyl ester (MAL) (ALA-based PDT) is approved for treatment of non-melanoma skin cancers including superficial basal cell carcinoma (BCC), actinic keratosis (AK), Bowen’s disease (BD), and squamous cell carcinoma in situ (SCC) in many countries [2]. In these cancers, ALA-based PDT may also be used in replacement or to reduce the extent of surgery [4]. Furthermore, the use of ALA-based PDT for the treatment of other types of cancer, e.g. in the brain, stomach and bladder, are currently being evaluated in clinical trials [3]. ALA-based PDT may also be used for the treatment of acne, psoriasis, scleroderma, viral warts, photoaging and cutaneous lymphoma [2].

ALA is an endogenous precursor of the potent photosensitizer protoporphyrin IX (PpIX), which is synthesized in the heme biosynthetic pathway of nucleated cells [5]. By administration of exogenous ALA the first rate-limiting step of the heme biosynthetic pathway, which is regulated by negative feedback of heme, is bypassed [6], [7]. Studies have furthermore indicated that PpIX accumulates in greater amounts in tumor cells than in normal cells following the administration of exogenous ALA [5]. The main reasons for the selective accumulation in cancer cells are the changes in the activity of two enzymes of the heme biosynthesis pathway, namely increased activity of porphoblinogen deaminase, which catalyzes an early step of the heme biosynthetic pathway, and decreased activity of ferrochelatase, catalyzing the conversion of PpIX to heme in the last step of the biosynthetic pathway [6]. MAL was developed to increase the hydrophobicity and hence skin penetration of the pro-drug. Once inside the cell, intracellular esterases catalyze the cleavage of the ALA esters to ALA, which then enters the heme biosynthetic pathway [7].

Due to the selective accumulation of PpIX in cancer cells, ALA-based PDT does not cause the serious adverse side effects often seen with conventional chemotherapy. The main limiting factor for successful clinical ALA-based PDT is pain, which in some cases is so severe that the treatment is discontinued [8], [9]. Although the mechanism of pain has not fully been elucidated, several studies have suggested that it may be due to nerve stimulation and tissue damage induced by ROS [10], [11]. Interestingly, clinical studies have shown that MAL may induce less pain than ALA [9], [12], [13].

Studies by our group and others have indicated that active cellular uptake of ALA is via γ-aminobutyric acid (GABA) transporters (GATs) [14]–[18], of which four human subtypes, GAT-1, GAT-2, GAT-3 and BGT-1 (betaine-GABA transporter-1), have been identified [19]-[22]. The uptake of MAL, however, seems to be cell type dependent [15]–[17]. In adenocarcinoma WiDr and LM3 cells, studies have indicated that MAL is transported via non-polar amino acid transporters rather than GAT [15], [18]. MAL uptake was also recently suggested to be via GATs and other amino acid transporters in rat peripheral DRG sensory neurons [16] and in human A431 and CCD skin cells [17].

The GATs belong to the neurotransmitter/sodium symporter (NSS) transporter family [23] of the solute carrier 6 (SLC6) superfamily [24]. The NSS family members mediate Na^+^-dependent uptake of a wide array of substrates, including dopamine (DAT), serotonin (SERT), noradrenaline (NET), glycine (GlyT) and GABA (GATs) [23], using an alternate access mechanism [25], [26]. During transport, the substrate binding site, which has a central location midway between the extracellular environment and the cytoplasm, is sequentially exposed to either side of the membrane through permeation pathways [25], [26]. Substrate transport thus involves cycling between outward-open, outward-occluded and inward-open conformational states of the transporters.

Only one member of the NSS family, namely the prokaryotic *Aquifex aeolicus* leucine transporter (LeuT), has so far been crystallized. In support of the alternate-access hypothesis, however, the LeuT crystal structures are available in outward-open, outward-occluded and inward-open conformations [27]–[29]. Co-crystallized with substrates the transporter is stabilized in an outward-occluded state [29]. In contrast, the crystal structure of LeuT in complex with the competitive inhibitor tryptophan (Trp) shows that Trp stabilizes LeuT in an outward-open conformation [29]. The LeuT crystal structures hence suggest that in order for transport to occur, the substrates must be able to induce a conformational change in the transporter from outward-open to outward-occluded.

In this study, homology models of the four human transporters in outward-occluded, outward- and inward-open conformations were constructed using three x-ray crystal structures of LeuT as templates [27]–[29]. To investigate the binding of GABA, ALA and MAL, the compounds were docked into in the central putative substrate binding sites in the outward-occluded GAT models. Furthermore, the electrostatic potentials (ESPs) of the putative translocation pathways leading from the extracellular environment to the central substrate binding site (termed the ‘entry’ pathway) and from the central substrate binding site to the cytoplasm (termed the ‘exit’ pathway) were calculated in the outward- and inward-open homology models, respectively. Our results suggest that whereas ALA most likely is a substrate of all four GAT subtypes, MAL may only be a substrate of GAT-2, GAT-3 and BGT-1. Furthermore, the results suggest that the major differences between the transporter subtypes most likely are located to the entry pathway. This region may hence be the most interesting to study with the aim of obtaining subtype-selective GAT inhibitors.

## Methods

### Homology Modeling

The amino acid sequences of GAT-1, GAT-2, GAT-3 and BGT-1 (UniProt accession numbers P30531, Q9NSD5, P48066 and P48065, respectively) [30] were aligned with LeuT using the Internal Coordinate Mechanics (ICM) version 3.7 software [31]. The alignment was adjusted according to the comprehensive alignment of prokaryotic and eukaryotic NSS transporter sequences published by Beuming *et al*. [32] (Figure S1).

Based on the alignment, outward-open GAT models were constructed using the 3F3A LeuT x-ray crystal structure [29] as template, while the outward-occluded and inward-open GAT models were generated based on the 2A65 [27] and 3TT3 [28] crystal structures, respectively. The ICM BuildModel macro was used to construct the models [31]. This macro uses a rigid body approach to transfer the conformation of the structurally conserved regions from the template to the target and constructs the non-conserved loop regions either by *ab initio* modeling (< seven amino acids) or by PDB loop searching (> seven amino acids) [31]. The final models consisted of the twelve TMs and the connecting intra- and extracellular loops, but did not include the N- and C-termini and parts of EL2.

The Na1 and Na2 sodium ions were copied into the outward-open and outward-occluded GAT models from their corresponding LeuT templates as the amino acids coordinating the two sodium ions are highly conserved [32]. Although Rud *et al.* [14] have suggested that three sodium ions are needed for the transmembrane transport of ALA, a Na^+^: Cl^-^ stoichiometry of 2∶1 was applied in this study due to the lack of positional knowledge of the putative third sodium ion. In addition, a chloride ion was placed in the position corresponding to the carboxylate carbon of LeuT amino acid E290 in the outward-open and outward-occluded models [33], [34].

### Energy Refinement

The ICM RefineModel macro (default settings) [35] was used to remove possible close contacts between amino acids in the models and to relax the structures. The macro performs 1) side chain sampling using the program module Montecarlo-fast [35], 2) iterative annealing with tethers, and 3) a second side chain sampling. Iterations of Montecarlo-fast consist of a random move followed by local energy minimization and the iteration is either accepted or rejected based on the energy [35].

### Structure Quality Check

The programs PROCHECK, ERRAT and VERIFY-3D, available through the Structural Analysis and Verification Server (SAVES, http://nihserver.mbi.ucla.edu/SAVES/), were used to perform structure quality checks of the models before and after the refinement step.

### Evaluation of the Outward-occluded Models by Docking

To evaluate whether the outward-occluded GAT models could separate binders from decoys, an evaluation test set containing 17 binders and 170 decoys was established (Figure S2; S3). The 170 decoys were selected using ICM Molcart [31] based on their structural similarities with the binders (Figure S3). The compounds were charge labeled using default ECEPP/3 partial charges [36] before docking.

The ICM PocketFinder macro (default settings) [37] was used to define the central putative substrate binding site of the outward-occluded GAT homology models into which the evaluation test set was docked. The PocketFinder algorithm uses the 3D protein structure to detect possible ligand binding pockets and does not require knowledge about potential ligands [37].

The test set database was docked using the ICM batch docking method and a semi-flexible docking approach in which the transporter, represented as 3D grid potential maps accounting for van der Waals (vdw), electrostatics, hydrophobic and hydrogen bonding interactions, was kept rigid while the ligands were fully flexible. ICM uses a Monte Carlo global energy optimization algorithm to dock the ligands [31]. Due to the stochastic nature of the docking procedure three parallel docking runs were performed.

The ICM VLS scoring function was used to score the resulting ligand-protein complexes. The scoring function uses steric, entropic, hydrogen bonding, hydrophobic and electrostatic terms to calculate the score and also include a correction term proportional to the number of atoms in the ligand to avoid bias towards larger ligands [38].

Following docking, receiver operating characteristic (ROC) curves for each GAT model were generated using the best scored orientation of each ligand from the three parallel docking simulations, and the normalized ‘area under curve’ (noAUC) value for each transporter was calculated.

### Full-atom Docking Refinement of ALA, MAL and GABA

Following semi-flexible docking of ALA, MAL and GABA, full-atom refinement of the complexes was performed. During the refinement, energy minimization and sampling of the side chain torsional angles of the amino acids within 5 Å of the ligands using ICM biased probability Monte Carlo (BPMC) [31] was performed. To score the complexes following the full-atom refinement, the ICM scanScoreExtrenal macro was used [35].

### Electrostatic Potentials (ESPs)

ICM PocketFinder [35] was used to detect the substrate translocation pathways in the outward-open and inward-open GAT models (Table S3; S4). The identified amino acids were selected and the ESPs of the amino acids were calculated using the ICM Rapid Exact-Boundary Electrostatics (REBEL) algorithm with a potential scale value of 5 kcal/e.u. charge units (default values) [31]. The Na1 and Na2 ions (with a charge of +1) were included in the ESP calculations in the outward-open homology model. The ESP of GABA, ALA and MAL were also calculated using the ICM-REBEL [31].

### Indexing of Residues

To facilitate comparison of amino acid positions between the four GAT subtypes, a generic numbering scheme developed for the NSS transporters [32], [39] is used in this paper. The most conserved residue in each of the twelve TM segments is given the number 50, and the other residues are numbered according to its position relative to this most conserved residue. Hence, a residue with a generic position number lower or higher than 50 indicates that it is located N- or C-terminal to the most conserved residue in the TM helix, respectively. The reference GAT-1 residues are as follows: W68^1.50^, P96^2.50^, Y140^3.50^, T217^4.50^, P247^5.50^, Q291^6.50^, F339^7.50^, F386^8.50^, Y432^9.50^, Y453^10.50^, P505^11.50^, and P549^12.50^ (Table S1).

## Results

### Homology Modeling

**Figure 1:**
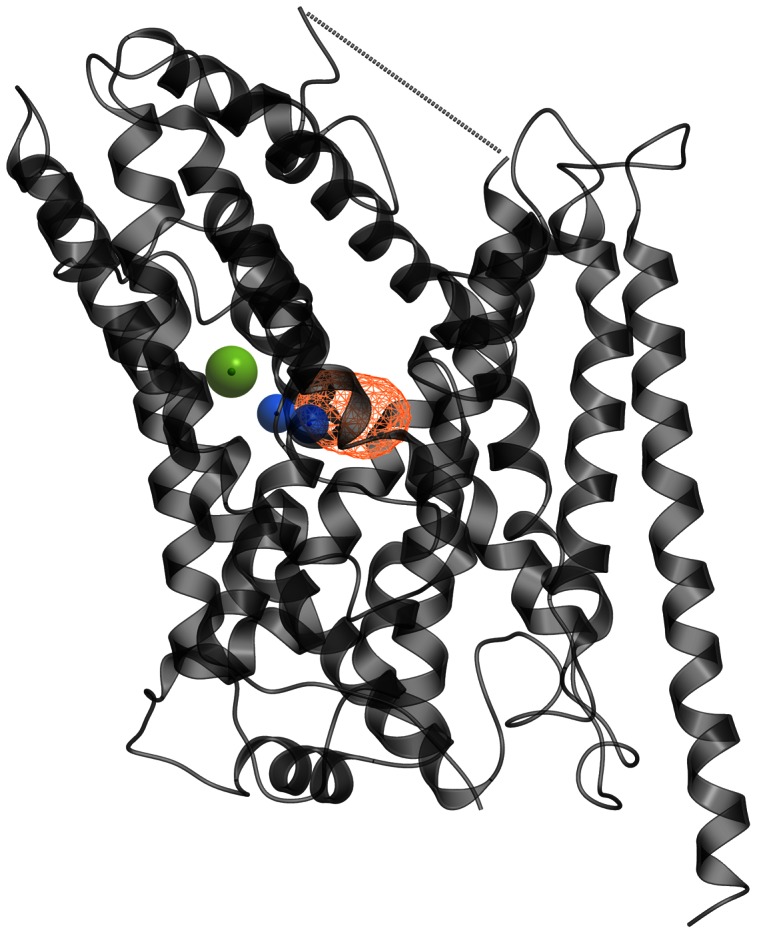
Outward-occluded GAT-2 model. Membrane view of the outward-occluded GAT-2 homology model (grey ribbon representation). Orange wire: the putative substrate binding site detected by ICM PocketFinder; blue spheres: Na1 and Na2 sodium ions; green sphere: chloride ion; dotted line: missing EL2 residues.

In this study, homology models of the four human GATs were constructed in outward-open, outward-occluded and inward-open conformations based on LeuT x-ray crystal structures (PDB id 3F3A, 2A65 and 3TT3, respectively). The stereochemical quality of the homology models both before and after energy refinements were evaluated using the PROCHECK [40], ERRAT [41] and VERIFY 3D [42], [43] programs and compared with LeuT template structures (Table S2). The ERRAT scores revealed that some atoms in the unrefined models had overlapping van der Waals surfaces, and these models were hence discarded. For the refined models, the ERRAT scores were similar to that of their corresponding templates and Ramachandran plots provided by the PROCHECK were found to be satisfactory (Table S2). The VERIFY 3D scores were lower for the refined structures than the corresponding LeuT, but acceptable (Table S2). The structure of the outward-occluded GAT-2 homology model is shown in Figure 1.

### Evaluation of the Outward-occluded Models by Docking

To further evaluate the outward-occluded models, an evaluation test set containing 17 binders and 170 decoys was docked into the central substrate binding site detected by ICM PocketFinder [35] (Table S3; S4). The compounds included as binders were either substrates or presumed substrates (i.e. compounds that only have been tested in some of the GATs but likely interact with all four transporter subtypes) and small-size inhibitors that presumably bind in the central substrate binding site (Figure S2). The decoys were selected based on their structural similarities with the binders (Figure S3). Specifically, all decoys contained at least one –COO^-^ or –SO_3_^-^ moiety.

The putative substrate binding site was formed by amino acids in TM 1, 3, 6, and 8 and the majority of the amino acids were conserved among the four GAT subtypes (Table 1). Some interesting differences were also seen between the transporters. For instance, GAT-2, GAT-3 and BGT-1 contained a negatively charged glutamate in position 1.42 which in GAT-1 was an aromatic tyrosine (Table 1). The GAT-1 pocket was identified as the largest of the four (Table 1). With the exception of L300^6.59^, all the identified amino acids have previously been shown by site-directed mutagenesis studies to play roles in the GABA binding and/or transport in GAT-1 [44], [45]. The localization of the putative GAT substrate binding site furthermore corresponded well to the position of the substrate binding site seen in the LeuT crystal structures and also with the results of Dodd and Christie [45]. Dodd and Christie showed that substitutions of the amino acids in positions 1.42, 3.46, 6.56 and 8.64 of the creatine transporter (CRT) to the corresponding GAT-1 amino acids results in the loss of creatine and gain of GABA transport activity [45].

**Table 1:** Central substrate binding site.

GAT-1	GAT-2	GAT-3	BGT-1	Position
Y60	E48	E66	E52	**1.42**
A61	I49	I67	I53	**1.43**
I62	*I*	*I*	*I*	**1.44**
G63	G51	G69	G55	**1.45**
L64	L52	L70	L56	**1.46**
G65	G53	G71	G57	**1.47**
N66	N54	*N*	*N*	**1.48**
L136	L125	L143	L129	**3.46**
Y140	Y129	Y147	Y133	**3.50**
F294	F288	F308	F293	**6.53**
S295	S289	S309	S294	**6.54**
G297	A291	A311	A296	**6.56**
L300	L294	L314	Q299	**6.59**
S396	S390	S410	S395	**8.60**
Q397	*Q*	*Q*	*Q*	**8.61**
C399	V393	V413	*V*	**8.63**
T400	C394	C414	C399	**8.64**
172.2	145.2	161.1	118.6	**Volume (Å^3^)**
156	140.4	150.3	121.4	**Area (Å^2^)**

Amino acids detected by ICM PocketFinder in the outward-occluded GAT models; in *italics*: amino acids not detected in the respective models.

**Figure 2:**
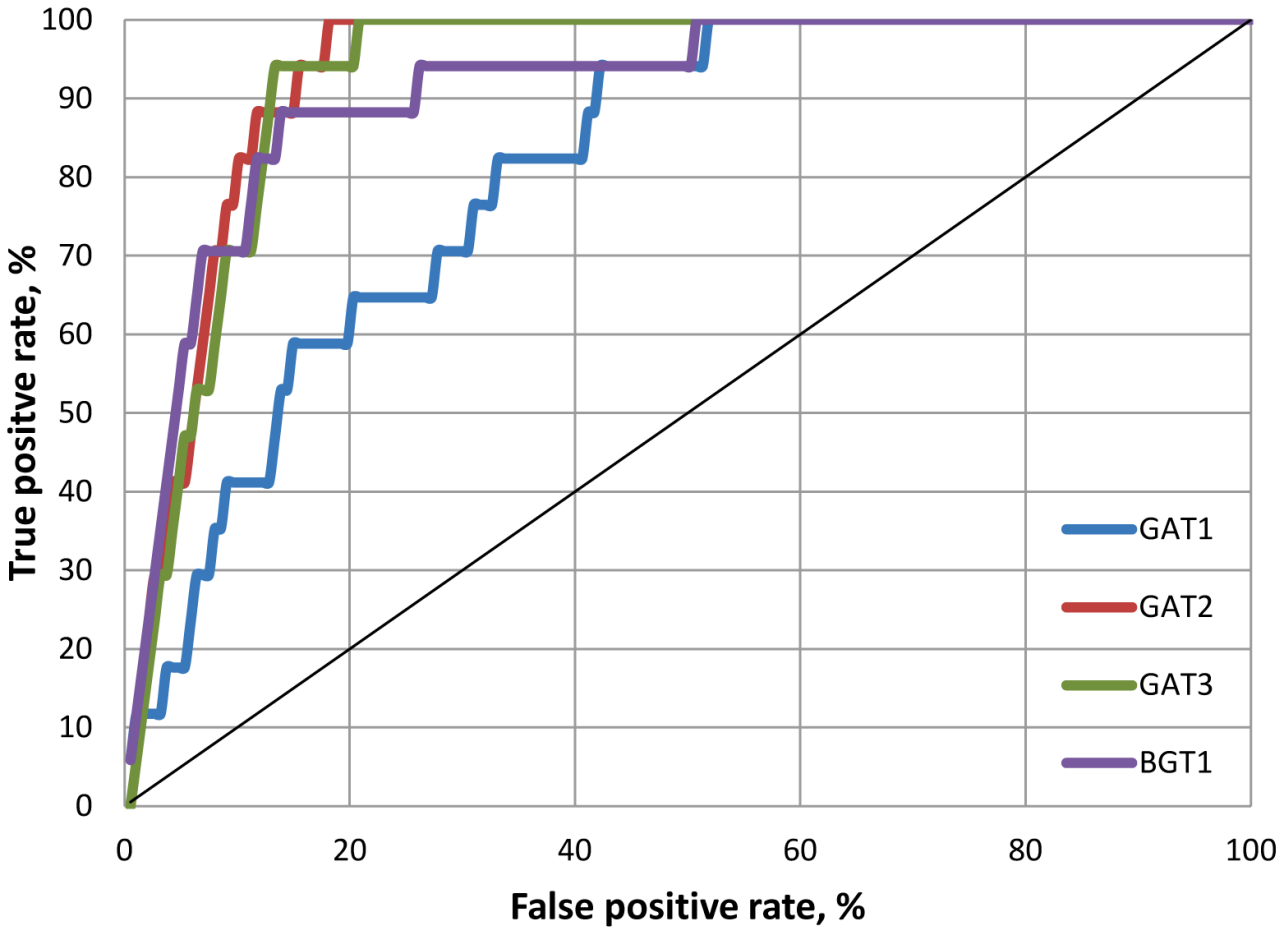
Evaluation docking results. ROC curves obtained from docking of 17 binders and 170 decoys into the central putative substrate binding sites of detected in the outward-occluded GAT models.

Following docking, ROC curves and noAUC values of each model were obtained (Figure 2). A noAUC value of 100 represents a perfect separation between binders and decoys. The results showed that the GAT-1 model was the least specific with a noAUC value of 68.8, whereas the noAUC values for other three subtypes were excellent (noAUC values of 95.7, 94.0 and 90.4 for GAT-2, GAT-3 and BGT-1, respectively) (Figure 2). Analysis of the docking results in GAT-1 showed that the lower noAUC value obtained for this transporter was due to more decoys rather than fewer substrates being selected. This was not surprising as the GAT-1 model had the largest binding pocket of the four models (Table 1). The evaluation docking hence suggested that the models were acceptable for docking of substrates.

### Docking of GABA, ALA and MAL

**Figure 3:**
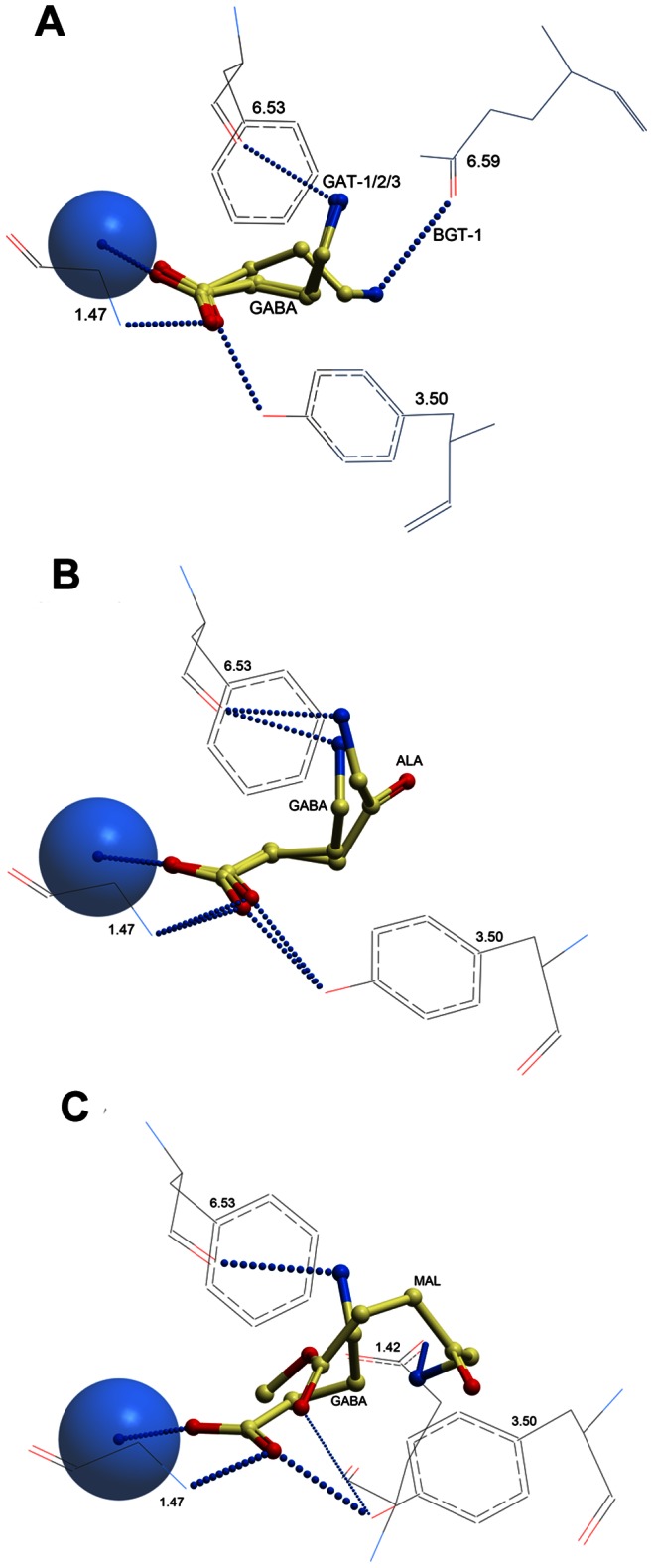
Orientations of GABA, ALA and MAL in the central substrate binding pocket. a) GABA in all four GAT models, b) GABA and ALA in GAT-2, and c) GABA and MAL in GAT-2. Amino acids in positions 1.47 (G), 3.50 (Y) and 6.53 (F) are conserved among the GAT subtypes, whereas the amino acids in positions 1.42 (Y in GAT-1; E in the others) and 6.59 (Q in BGT-1, L in the others) are non-conserved. Intermolecular hydrogen bonds are shown as dotted lines; the thickness of the lines representing the energy of the interaction. Amino acid side chains are shown in wire representation, ligands in yellow xstick representation, and Na1 sodium ion as blue sphere. Color coding of atoms: blue: nitrogen; red: oxygen.

To study the interaction of PDT pro-drugs ALA and MAL and the native substrate GABA in the GAT models, the ligands were docked into the central putative substrate binding site of the outward-occluded GAT models using a regular semi-flexible docking approach, followed by refinement of the GAT substrate binding site amino acids within 5 Å of the three ligands. The results showed that GABA, ALA and MAL had favorable (i.e. negative) docking scores in all four GATs, except MAL in GAT-1 (Table 2). The orientations of GABA, ALA and MAL in the central substrate binding site can be seen in Figure 3.

**Table 2:** Docking scores (kcal/mol) of GABA, ALA and MAL.

Ligand	GAT-1	GAT-2	GAT-3	BGT-1
GABA	−30.12	−28.37	−20.51	−36.14
ALA	−32.10	−23.40	−27.62	−35.77
MAL	7.11	−19.10	−19.72	−19.00

The docking results showed that the carboxyl group of GABA coordinated the Na1 ion and formed hydrogen bonds to the side chain hydroxyl group of Y^3.50^ and to the main chain nitrogen atom of G^1.47^ in all four GAT subtypes (Figure 3). The amine moiety of GABA formed a hydrogen bond to the main chain oxygen of F^6.53^ in GAT-1, GAT-2 and GAT-3, whereas it in BGT-1 was involved in a hydrogen bond to the side chain oxygen of Q^6.59^, which is a leucine residue in the other GATs (Table 1).

The GABA orientations are in accordance with the results of docking of GABA in GAT-1, GAT-2 and GAT-3 published by other groups, in which the orientation of the carboxyl moiety of GABA was very similar to the present orientation whereas the localization of the amine moiety was more variable [46]–[49]. This was not surprising as the same template was used for homology modeling in all studies, and comparison of GABA in the GAT models and Leu in the template structure showed that GABA occupied the same regions of the binding pocket as Leu in the template structure [27] (results not shown).

The carboxyl moiety of ALA had a similar orientation as that of GABA, interacting with Na1, Y^3.50^ and G^1.47^ (Figure 3). Furthermore, like GABA, the amine moiety in ALA was found in two localizations in the transporter models: in GAT-1 and GAT-2 the amine moiety interacted with the backbone oxygen atom of F^6.53^, whereas it in GAT-3 and BGT-1 the moiety formed an ionic interaction with the side chain of E^1.42^ (which is a tyrosine in GAT-1) (Figure 3).

MAL occupied the same region as GABA and ALA in the GATs (Figure 3). However, as MAL contains an ester moiety whereas GABA and ALA have a carboxylate moiety, MAL was not able to coordinate the Na1 ion (Figure 3). In GAT-1, a hydrogen bond was formed between the ester and amine moieties of the ligand and to the backbone oxygen atom of F^6.53^ (results not shown). In GAT-2, GAT-3 and BGT-1, hydrogen bonds were present between the ester and amine moieties of MAL and between the ester moiety and the side chain hydroxyl of Y^3.50^ (Figure 3). Furthermore, in the latter transporters, the amine moiety of MAL in addition formed ionic interactions with E^1.42^ (Figure 3), while the corresponding amino acid in GAT-1 was tyrosine (Table 1). The ionic interaction with E^1.42^ probably accounted for the relatively high scoring of MAL in GAT-2, GAT-3 and BGT-1 (Table 2).

### Electrostatic Potentials (ESP) of Outward- and Inward-open Homology Models

**Figure 4:**
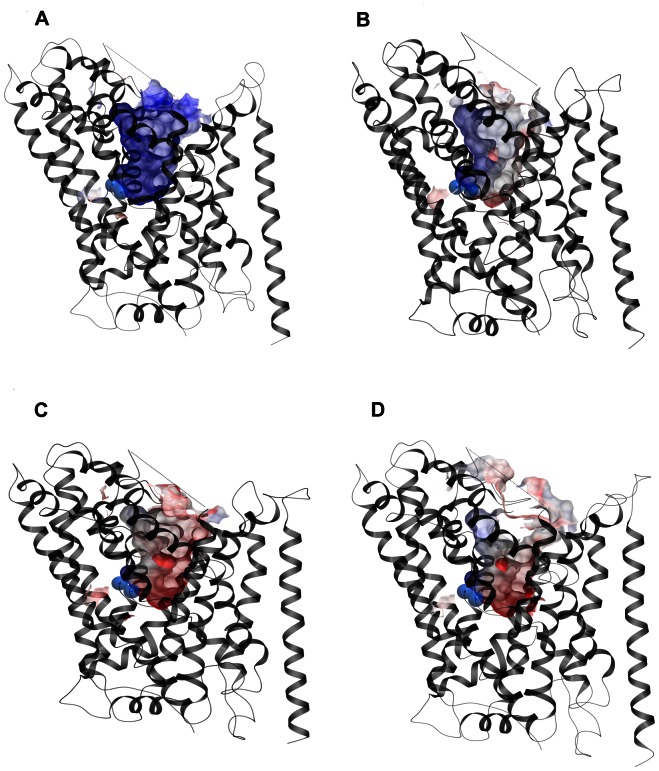
Entry pathway ESPs. ESPs of the entry pathways detected in the outward-open GAT models (grey ribbon representation). a) GAT-1, b) GAT-2, c) GAT-3, and d) BGT-1. Blue spheres: Na1 and Na2 sodium ions. Color coding: red: negative ESP; blue: positive ESP; grey: neutral ESP.

**Figure 5:**
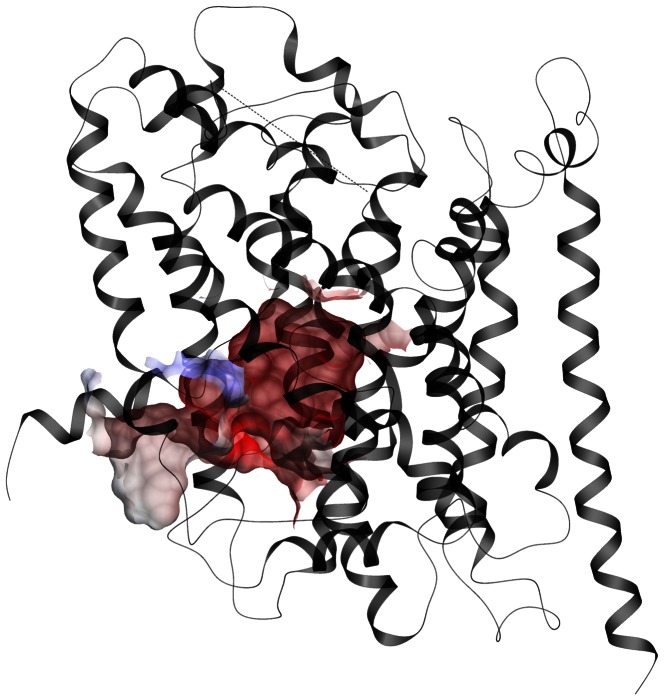
GAT-1 exit pathway ESPs. GAT-1 in grey ribbon representation. Color coding of ESPs as in Figure 4.

**Figure 6:**
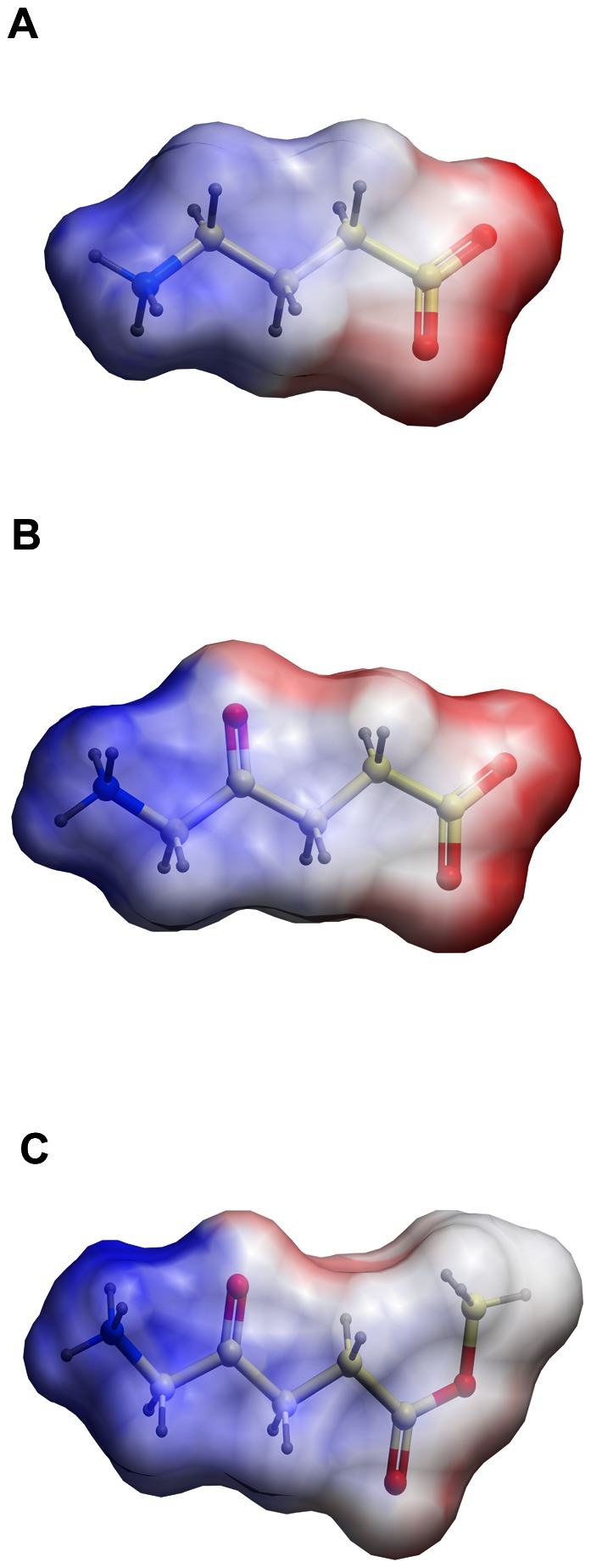
GABA (a), ALA (b) and MAL (c) ESPs. **Color coding of ESPs as in **Figure 4****.****

The ESPs of the funnel-shaped entry pathway extending from the extracellular environment to the central substrate binding pocket in the outward-open homology models varied considerably (Figure 4). Whereas the GAT-1 entry pathway and central putative substrate binding site was highly positive in nature, the corresponding areas in GAT-2, GAT-3 and BGT-1 consisted of positive, negative and hydrophobic sub-regions (Figure 4). The major differences between the GAT subtypes in the entry pathway were the amino acids in position 1.42, 6.59 and 8.64, located in the central substrate binding site region, and the amino acids in positions 1.54, EL4 and 10.45, located in the vestibule leading from the extracellular environment to the central substrate binding site (Table S3; S4). In contrast, only minor differences in the ESPs of the exit pathway reaching from the central substrate binding site to the cytoplasm in the inward-open GAT models were observed, and this region was highly negative in all four GAT subtypes (Figure 5). The ligand ESPs indicated that the surface of MAL is more positively charged than that of GABA and ALA which had zwitterionic charge distribution (Figure 6).

## Discussion

Whereas studies have suggested that the PDT pro-drug ALA is a GAT substrate [16], [17], studies regarding MAL are more ambiguous as this compound seemingly is transported via GAT in some cell types but not in others [15], [17]. Molecular insight into the binding interactions of GABA, ALA and MAL in the central substrate binding site of the four GAT subtypes may help shed light on this question.

GABA is the primary inhibitory neurotransmitter in the central nervous system (CNS) and a native substrate for the GATs. The GATs play an essential role in regulating neurotransmitter signaling and homeostasis by mediating uptake of released GABA from the extracellular space into neurons and glial cells. Abnormal levels of GABA can result in inappropriate neural signaling and underlie CNS disorders such as epilepsy, depression, schizophrenia, drug addiction, and acute and chronic pain [50]–[54]. GAT-1 for instance plays an important role in the treatment of epilepsy being targeted by the antiepileptic tiagabine [55]. The GATs may, however, also play important roles in non-CNS and non-neuronal diseases. In contrast to GAT-1, which is exclusively expressed in the CNS, GAT-2 and BGT-1 are also expressed in the peripheral nervous system (PNS), and has been found in several other tissues, including the kidneys, liver, heart, lungs, and testis [21], [22], [27], [56]. GAT-3 was also recently shown to be expressed in human skin cells [17]. Molecular insight into the structure and function of the GATs is important for an increased understanding of GABAergic neurotransmission and may be important for drug development in several therapeutic areas.

In the present study, the outward-occluded GAT models, in which the central substrate binding site is closed from either side of the membrane, were chosen for docking of the native substrate GABA and the putative substrates ALA and MAL as x-ray crystal structures show that LeuT in the presence of substrates adopts this conformation [29]. Based on the orientations of GABA, ALA and MAL in the substrate binding site, as well as the docking scores, our results suggest that ALA may be a substrate in all four GATs whereas MAL may be a substrate in GAT-2, GAT-3 and BGT-1. However, whether a compound is transported or not via GAT is also dependent on other factors than the ability to bind to and induce the outward-occluded conformation of the transporter. Dodd and Christie have for instance shown that though the creatine transporter activity can be changed from creatine to GABA by substitution of a few amino acids in the central substrate binding site, the substitutions alone are not sufficient for efficient GABA transport [45]. Hence, though the obtained docking orientations and scores suggested that ALA and MAL may be substrates of all or some GAT subtypes, further studies are needed to verify these findings.

The ESPs of the translocation pathways may reveal electrostatic forces involved in substrate binding and translocation and highlight differences between the four GAT subtypes. The ESPs of the putative entry and exit permeation pathways in the outward- and inward-open GAT homology models, respectively, were hence calculated (Figure 4; 5). The x-ray structure of LeuT in complex with the competitive inhibitor tryptophan (Trp) [29] shows that Trp prevents the extracellular gate from closing, hence stabilizing the transporter in a conformation in which the central substrate binding site is accessible from the extracellular environment [29]. The outward-open GAT models constructed based on this LeuT structure were hence used to illustrate the entry pathways. The inward-open LeuT crystal structure was used as a template for modeling the GAT subtypes used to calculate the ESPs of the exit pathway extending from the central substrate binding site to the cytoplasm. In this structure, the extracellular gate has closed, an intracellular vestibule has opened and the Na1 and Na2 sodium binding sites seen in the outward-open and outward-occluded structures have been disrupted [28]. These changes has occurred due to large conformational changes, including reorientation of TMs 1, 2, 5, 6 and 7, hinge bending of the intracellular half of TM1 and occlusion of the extracellular vestibule by EL4 [28].

The ESP calculations indicated that the major differences between the GAT subtypes were located in the outward-open models, hence in the entry pathway region of the transporters (Figure 4). The ligand ESPs also showed that GABA and ALA had a zwitterionic charge distribution, whereas the MAL charge distribution was cationic in nature due to the replacement of the carboxyl moiety found in GABA and ALA with an ester group (Figure 6). The ESPs hence support the notion that MAL may not be a GAT-1 substrate, as the results suggest that the entry pathway of this GAT subtype is highly positive in nature (Figure 4).

The amino acids in the entry region are the first to come in contact with the substrates and hence play crucial roles in ligand recognition and binding. The finding that the major differences between the GAT subtypes are located in this region may be of clinical importance as it has been suggested that the pain often observed during ALA-based PDT may result from uptake of ALA via GAT-2 and BGT-1 into the mitochondria-rich sensory neurons and hence high-level accumulation of PpIX [11], [57]. Current pain-reducing strategies include interrupted illumination, cooling of the affected area and local anesthesia [58], [59]; however, in some cases the pain is severe and the treatment is discontinued [8], [9]. Exploitation of the differences in the entry pathways to develop inhibitors that can be used to selectively inhibit the uptake of ALA into the sensory neurons may hence be used clinically to reduce ALA-induced pain.

In summary, this study pioneers in structure-based characterization of ALA and MAL transports via the four GABA transporters using the homology modeling approach. Although ALA-based PDT has been used successfully for the treatment of a variety of skin cancers, pain is a limiting factor. ALA-based PDT in combination with selective inhibitors of the GAT may be an attractive approach to develop pain-reduce strategy and improve the PDT efficacy in the future.

## Supporting Information

Figure S1 Alignment.
(TIF)

Figure S2 Evaluation test set binder structures.
(TIF)

Figure S3 Evaluation test set decoy structures.
(TIF)

Table S1 NSS numbering scheme.
(DOCX)

Table S2 SAVES results.
(DOCX)

Table S3 Amino acids, entry pathway.
(DOCX)

Table S4 Amino acids, exit pathway. The GAT homology models constructed in this study are available from the authors upon request.
(DOCX)
