# Supplementary figures and images for: Homology Modeling of Human γ-Butyric Acid Transporters and the Binding of Pro-Drugs 5-Aminolevulinic Acid and Methyl Aminolevulinic Acid Used in Photodynamic Therapy
Source: PLoS One. 2013 Jun 7;8(6):e65200. doi: 10.1371/journal.pone.0065200 (PMC3676387; doi:10.1371/journal.pone.0065200)

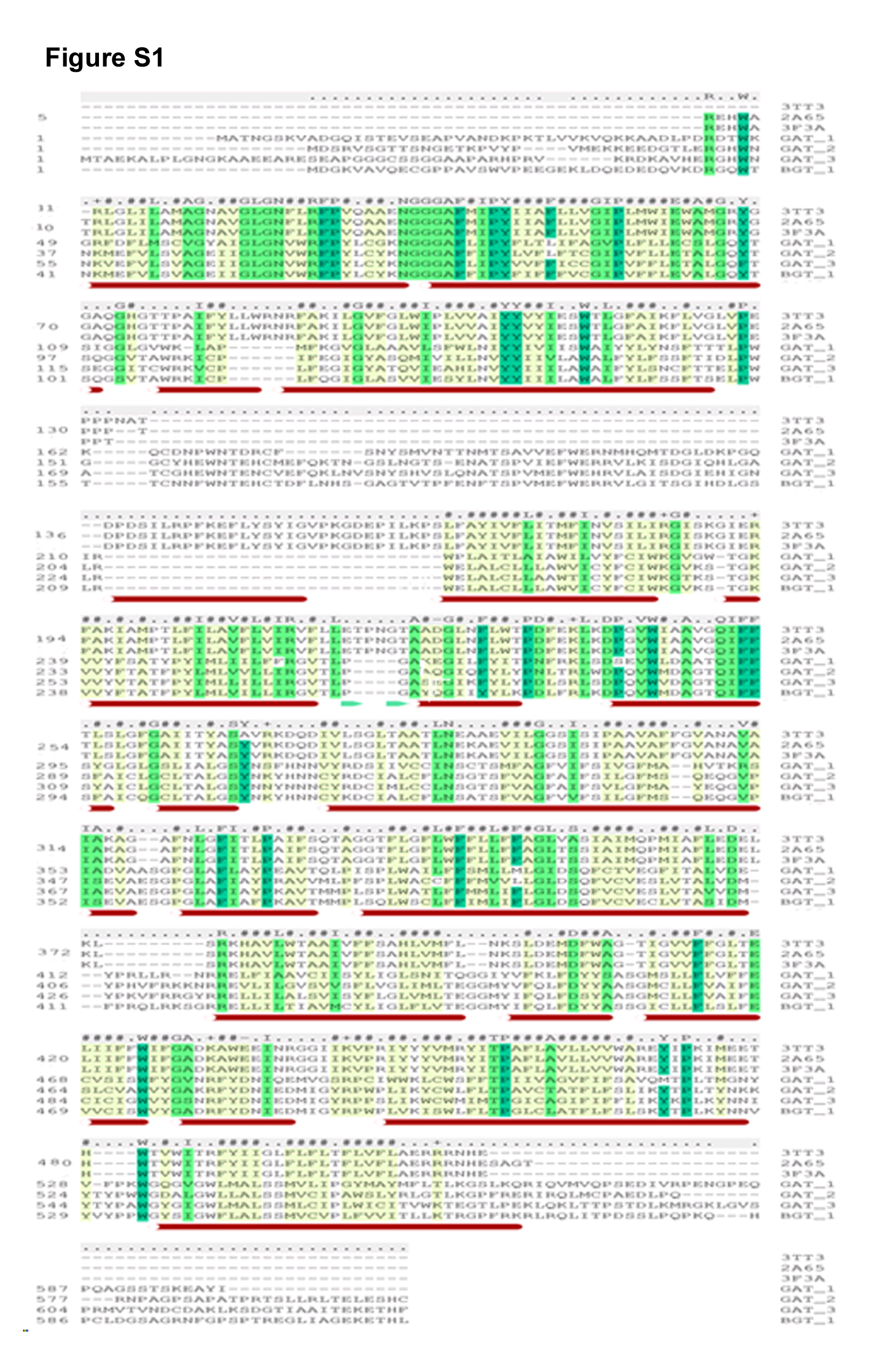

Supplement: Figure S1 — Alignment. (TIF) [file pone.0065200.s001.tif]

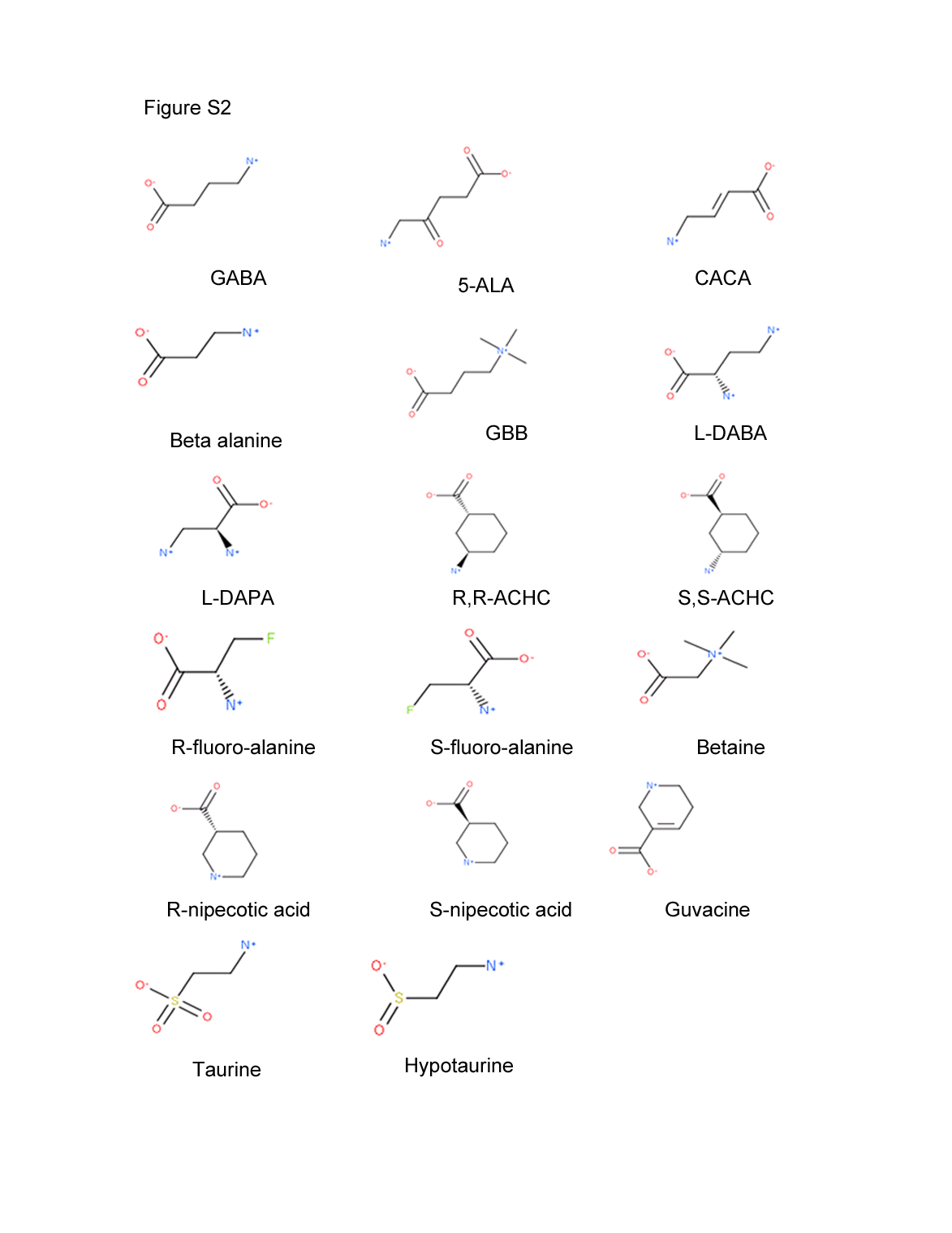

Supplement: Figure S2 — Evaluation test set binder structures. (TIF) [file pone.0065200.s002.tif]

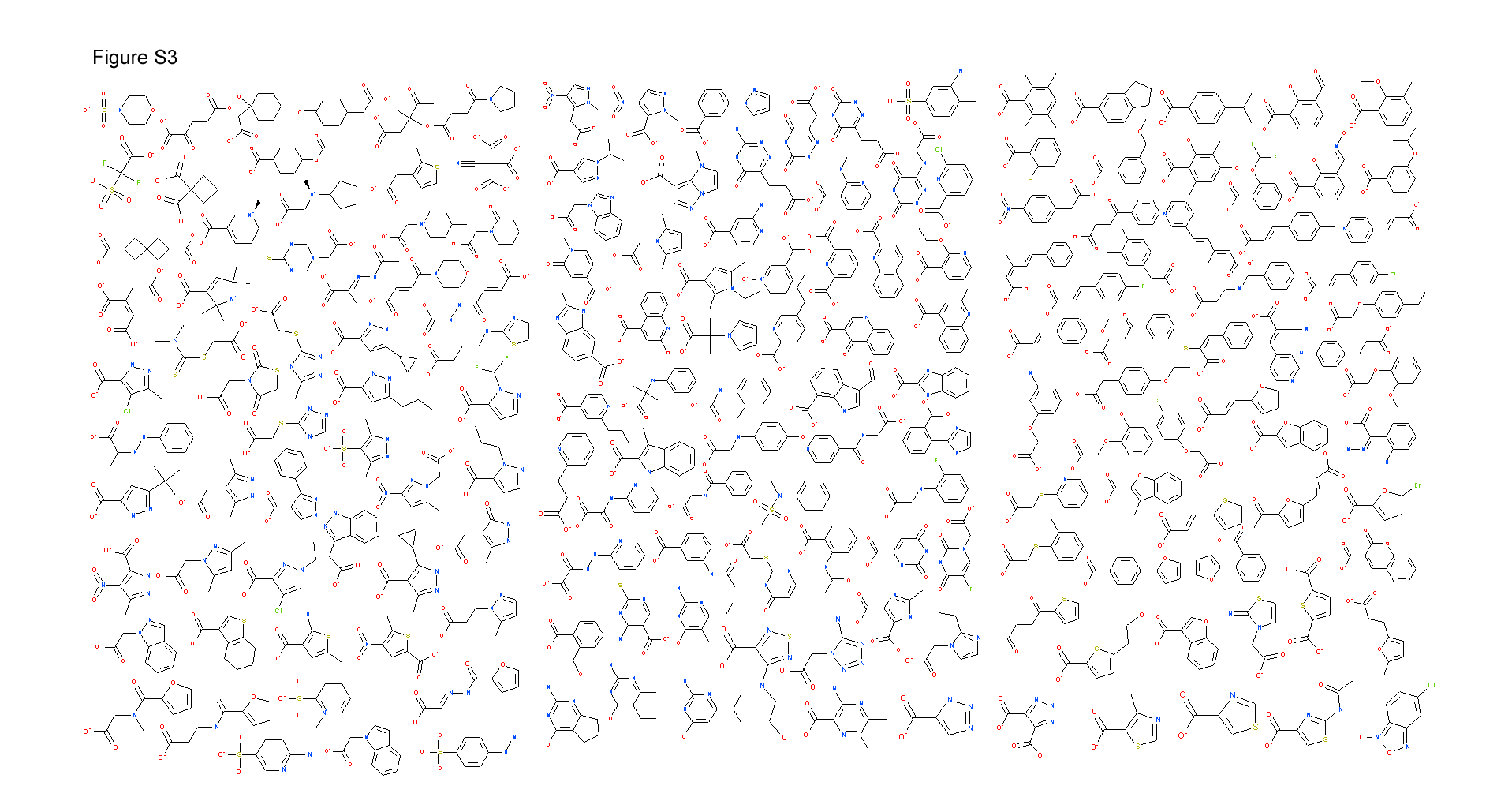

Supplement: Figure S3 — Evaluation test set decoy structures. (TIF) [file pone.0065200.s003.tif]
