# Supplementary material for: Homology Modeling of Human γ-Butyric Acid Transporters and the Binding of Pro-Drugs 5-Aminolevulinic Acid and Methyl Aminolevulinic Acid Used in Photodynamic Therapy
Source: PLoS One. 2013 Jun 7;8(6):e65200. doi: 10.1371/journal.pone.0065200 (PMC3676387; doi:10.1371/journal.pone.0065200)
Supplement: Table S1 — NSS numbering scheme. (DOCX) [file pone.0065200.s004.docx]

**Table S1.**

| **Position** | **LeuT** | **GAT-1** | **GAT-2** | **GAT-3** | **BGT-1** |
| --- | --- | --- | --- | --- | --- |
| 1.50 | L29 | W68 | W56 | W74 | W60 |
| 2.50 | P57 | P96 | P84 | P102 | P88 |
| 3.50 | Y108 | Y140 | Y129 | Y147 | Y133 |
| 4.50 | V171 | T217 | C211 | C231 | C216 |
| 5.50 | L202 | P247 | P241 | P261 | P246 |
| 6.50 | Q250 | Q291 | Q285 | Q305 | Q290 |
| 7.50 | S298 | F339 | F333 | F353 | F338 |
| 8.50 | F345 | F386 | F379 | F400 | F385 |
| 9.50 | F387 | Y432 | F428 | Y448 | Y433 |
| 10.50 | W406 | Y453 | Y449 | Y469 | Y454 |
| 11.50 | P457 | P505 | P501 | P521 | P506 |
| 12.50 | T498 | P549 | P546 | P566 | P551 |

**Table S1**. NSS numbering scheme.
