# Supplementary material for: Homology Modeling of Human γ-Butyric Acid Transporters and the Binding of Pro-Drugs 5-Aminolevulinic Acid and Methyl Aminolevulinic Acid Used in Photodynamic Therapy
Source: PLoS One. 2013 Jun 7;8(6):e65200. doi: 10.1371/journal.pone.0065200 (PMC3676387; doi:10.1371/journal.pone.0065200)
Supplement: Table S2 — SAVES results. (DOCX) [file pone.0065200.s005.docx]

**Table S2**.

|  | **Template** | **Template** | **Template** |
| --- | --- | --- | --- |
| **GAT-1** | **2A65** | **3F3A** | **3TT3** |
| Ramachandran plot | 95.3 / 4.7 / 0 | 94.7 / 5.3 / 0 | 95.5 / 4.2 / 0.3 |
| Errat | 87.931 | 92.391 | 86.726 |
| Verify-3D | 75.26 % | 77.40 % | 74.51 % |
| **GAT-2** | **2A65** | **3F3A** | **3TT3** |
| Ramachandran plot | 94.2 / 5.5 / 0.3 | 94.8 / 5.2 / 0 | 95.5 / 3.9 / 0.6 |
| Errat | 92.308 | 94.828 | 89.956 |
| Verify-3D | 81.55 % | 78.86 % | 67.02 % |
| **GAT-3** | **2A65** | **3F3A** | **3TT3** |
| Ramachandran plot | 94.2 / 5.5 / 0.3 | 94.7 / 5.3 / 0 | 94.9 / 4.5 / 0.6 |
| Errat | 93.162 | 87.931 | 90.749 |
| Verify-3D | 79.25 % | 79.92 % | 71.73 % |
| **BGT-1** | **2A65** | **3F3A** | **3TT3** |
| Ramachandran plot | 94.0 / 6.0 / 0 | 94.8 / 5.2 / 0 | 94.5 / 4.7 / 0.8 |
| Errat | 92.949 | 93.966 | 88.210 |
| Verify-3D | 83.23 % | 78.22 % | 67.88 % |
| **LeuT** | **2A65** | **3F3A** | **3TT3** |
| Ramachandran plot | 94.5 / 5.5 / 0 | 94.9 / 5.1 / 0 | 92.3 / 6.7 / 0 |
| Errat | 93.028 | 94.400 | 87.889 |
| Verify-3D | 93.15 % | 90.18 % | 90.06 % |

**Table S2**. SAVES results. Ramachandran plot: most favored/additional allowed/generously allowed regions.
