# Supplementary material for: Homology Modeling of Human γ-Butyric Acid Transporters and the Binding of Pro-Drugs 5-Aminolevulinic Acid and Methyl Aminolevulinic Acid Used in Photodynamic Therapy
Source: PLoS One. 2013 Jun 7;8(6):e65200. doi: 10.1371/journal.pone.0065200 (PMC3676387; doi:10.1371/journal.pone.0065200)
Supplement: Table S3 — Amino acids, entry pathway. (DOCX) [file pone.0065200.s006.docx]

**Table S5.**

| GAT-1 | GAT-2 | GAT-3 | BGT-1 | Position |
| --- | --- | --- | --- | --- |
| Y60 | E48 | E66 | E52 | **1.42** |
| A61 | I49 | I67 | I53 | **1.43** |
| I62 | I50 | I68 | I54 | **1.44** |
| G63 | G51 | G69 | G55 | **1.45** |
| L64 | L52 | L70 | L56 | **1.46** |
| G65 | G53 | G71 | G57 | **1.47** |
| N66 | N54 | N72 | N58 | **1.48** |
| W68 | W56 | W74 | W60 | **1.50** |
| R69 | R57 | R75 | R61 | **1.51** |
| Y72 | Y60 | R78 | R64 | **1.54** |
| L136 | L125 | L143 | L129 | **3.46** |
| Y139 | Y128 | Y146 | Y132 | **3.49** |
| Y140 | Y129 | Y147 | Y133 | **3.50** |
| I143 | V132 | I150 | I136 | **3.53** |
| W146 | W135 | W153 | W139 | **3.56** |
| F294 | F288 | F308 | F293 | **6.53** |
| S295 | S289 | S309 | S294 | **6.54** |
| Y296 | F290 | Y310 | F295 | **6.55** |
| G297 | A291 | A311 | A296 | **6.56** |
| L300 | L294 | L314 | Q299 | **6.59** |
| A357 | A351 | A371 | A356 | **EL4** |
| A358 | E352 | E372 | E358 | **EL4** |
| G360 | G354 | G374 | G361 | **EL4** |
| P361 | P355 | P375 | P362 | **EL4** |
| L363 | L357 | L377 | L364 | **EL4** |
| A364 | A358 | A378 | A365 | **EL4** |
| F365 | F359 | F379 | F366 | **EL4** |
| S396 | S390 | S410 | S395 | **8.60** |
| T400 | C394 | C414 | C399 | **8.64** |
| F447 | F443 | F463 | F448 | **10.44** |
| K448 | Q444 | Q464 | Q449 | **10.45** |
| D451 | D447 | D467 | D452 | **10.48** |
| L460 | L456 | L476 | L461 | **10.57** |

**Table S5.** Amino acids of the entry pathway, outward-open models.
