# Supplementary material for: Homology Modeling of Human γ-Butyric Acid Transporters and the Binding of Pro-Drugs 5-Aminolevulinic Acid and Methyl Aminolevulinic Acid Used in Photodynamic Therapy
Source: PLoS One. 2013 Jun 7;8(6):e65200. doi: 10.1371/journal.pone.0065200 (PMC3676387; doi:10.1371/journal.pone.0065200)
Supplement: Table S4 — Amino acids, exit pathway. The GAT homology models constructed in this study are available from the authors upon request. (DOCX) [file pone.0065200.s007.docx]

**Table S6**.

| GAT-1 | GAT-2 | GAT-3 | BGT-1 | Position |
| --- | --- | --- | --- | --- |
| S56 | S44 | S62 | S48 | 1.38 |
| G59 | G47 | G65 | G51 | 1.41 |
| Y60 | E48 | E66 | E52 | 1.42 |
| A61 | I49 | I67 | I53 | 1.43 |
| I62 | I50 | I68 | I54 | 1.44 |
| G63 | G51 | G69 | G55 | 1.45 |
| L64 | L52 | L70 | L56 | 1.46 |
| G65 | G53 | G71 | G57 | 1.47 |
| N66 | N54 | N72 | N58 | 1.48 |
| F98 | F86 | F104 | F90 | 2.48 |
| L136 | L125 | L143 | L129 | 3.46 |
| Y140 | Y129 | Y147 | Y133 | 3.50 |
| V240 | V234 | V254 | V239 | 5.43 |
| Y241 | Y235 | Y255 | Y240 | 5.44 |
| S243 | T237 | T257 | T242 | 5.46 |
| A244 | A238 | A258 | A243 | 5.47 |
| T245 | T239 | T259 | T244 | 5.48 |
| F294 | F288 | F308 | F293 | 6.53 |
| S295 | S289 | S309 | S294 | 6.54 |
| Y296 | F290 | Y310 | F295 | 6.55 |
| G297 | A291 | A311 | A296 | 6.56 |
| L300 | L294 | L314 | Q299 | 6.59 |
| S302 | C296 | C316 | C301 | 6.61 |
| L303 | L297 | L317 | L302 | 6.62 |
| L306 | L300 | L320 | L305 | 6.65 |
| V323 | L317 | L337 | L322 | 7.34 |
| N327 | N321 | N341 | N326 | 7.38 |
| M391 | L385 | F405 | F390 | 8.55 |
| L392 | L386 | L406 | L391 | 8.56 |
| D395 | D389 | D409 | D394 | 8.59 |
| S396 | S390 | S410 | S395 | 8.60 |
| F398 | F392 | F412 | F397 | 8.62 |
| C399 | V393 | V413 | V398 | 8.63 |
| T400 | C394 | C414 | C399 | 8.64 |

**Table S6**. Amino acids of the exit pathway, inward-open homology models.
